# Deciphering the secret codes in N^7^‐methylguanosine modification: Context‐dependent function of methyltransferase‐like 1 in human diseases

**DOI:** 10.1002/ctm2.70240

**Published:** 2025-02-20

**Authors:** Huan Fang, Jing He, Dan Du, Xue Wang, Xinyu Xu, Linping Lu, Yefan Zhou, Yangyang Wen, Fucheng He, Yingxia Li, Hongtao Wen, Mingxia Zhou

**Affiliations:** ^1^ Department of Gastroenterology The First Affiliated Hospital of Zhengzhou University Zhengzhou Henan China; ^2^ Department of Breast Surgery The First Affiliated Hospital of Zhengzhou University Zhengzhou Henan China; ^3^ Department of Medical Laboratory The First Affiliated Hospital of Zhengzhou University Zhengzhou Henan China

**Keywords:** cancer progression, METTL1, N^7^‐methylguanosine (m^7^G), RNA modification, therapeutic potential

## Abstract

N^7^‐methylguanosine (m^7^G) is one of the most prevalent post‐transcriptional modifications of RNA and plays a critical role in RNA translation and stability. As a pivotal m^7^G regulator, methyltransferase‐like 1 (METTL1) is responsible for methyl group transfer during the progression of m^7^G modification and contributes to the structure and functional regulation of RNA. Accumulating evidence in recent years has revealed that METTL1 plays key roles in various diseases depending on its m^7^G RNA methyltransferase activity. Elevated levels of METTL1 are typically associated with disease development and adverse consequences. In contrast, METTL1 may act as a disease suppressor in several disorders. While the roles of m^7^G modifications in disease have been extensively reviewed, the critical functions of METTL1 in various types of disease and the potential targeting of METTL1 for disease treatment have not yet been highlighted. This review describes the various biological functions of METTL1, summarises recent advances in understanding its pathogenic and disease‐suppressive functions and discusses the underlying molecular mechanisms. Given that METTL1 can promote or inhibit disease processes, the possibility of applying METTL1 inhibitors and agonists is further discussed, with the goal of providing novel insights for future disease diagnosis and potential intervention targets.

**Key points:**

- METTL1‐mediated m7G modification is crucial for various biological processes, including RNA stability, maturation and translation.
- METTL1 has emerged as a critical epigenetic modulator in human illnesses, with its dysregulated expression correlating with multiple diseases progression and presenting opportunities for both diagnostic biomarker development and molecular‐targeted therapy.
- Enormous knowledge gaps persist regarding context‐dependent regulatory networks of METTL1 and dynamic m7G modification patterns, necessitating mechanistic interrogation to bridge basic research with clinical translation in precision medicine.

## Graphical abstract

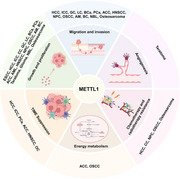

## 1 BACKGROUND

Epigenetics encompasses heritable structural and biochemical alterations of the chromatin without changing the nucleotide sequence.^1^ Epigenetic mechanisms manipulate various physiological and pathological processes through regulation of expressions of the relevant genes by changing the accessibility of epigenetic codes to chromatin locally and globally.^2^, ^3^, ^4^ Some epigenetic codes, including DNA methylation, histone modifications, chromatin remodelling and RNA modifications, have been extensively studied.^5^, ^6^ Epigenetic modifications of RNAs affect all RNA processes, including splicing, stability, folding, transportation and localisation.^7^, ^8^, ^9^ More than 170 types of chemical modifications have been discovered in various types of RNAs, including N^6^‐methyladenosine, N^6^, 2′‐O‐dimethyladenosine, N^1^‐methyladenosine, 5‐methylcytosine, 5‐hydroxymethylcytosine, N^4^‐acetylcytidine, pseudouridine and N^7^‐methylguanosine (m^7^G) modifications.^10^, ^11^ The m^7^G modification is one of the most common RNA modifications and plays a critical role in cancer biology by impacting RNA translation and stability.^12^

Methyltransferase‐like 1 (METTL1) was identified as one of the main m^7^G methyltransferases.^13^ METTL1 plays a role in several biological functions, including cell cycle progression, migration, invasion, angiogenesis, chemo‐ and radiotherapy resistance, energy metabolism and tumour immune microenvironment (TIME) suppression. In addition, numerous studies have shown that METTL1 regulates the initiation and progression of various diseases that are tightly dependent on the m^7^G methylation modification, which may be used as a biomarker or a new candidate target for the early diagnosis and treatment of diseases.^12^, ^14^ In this review, we describe the biological functions of METTL1 in detail. Then, we focus on the specific mechanisms of METTL1 in disease occurrence and development and discuss its relationship with the treatment efficiency and prognosis of disease.

## 2 m^7^G MODIFICATION AND ITS FUNCTION

**FIGURE 1:**
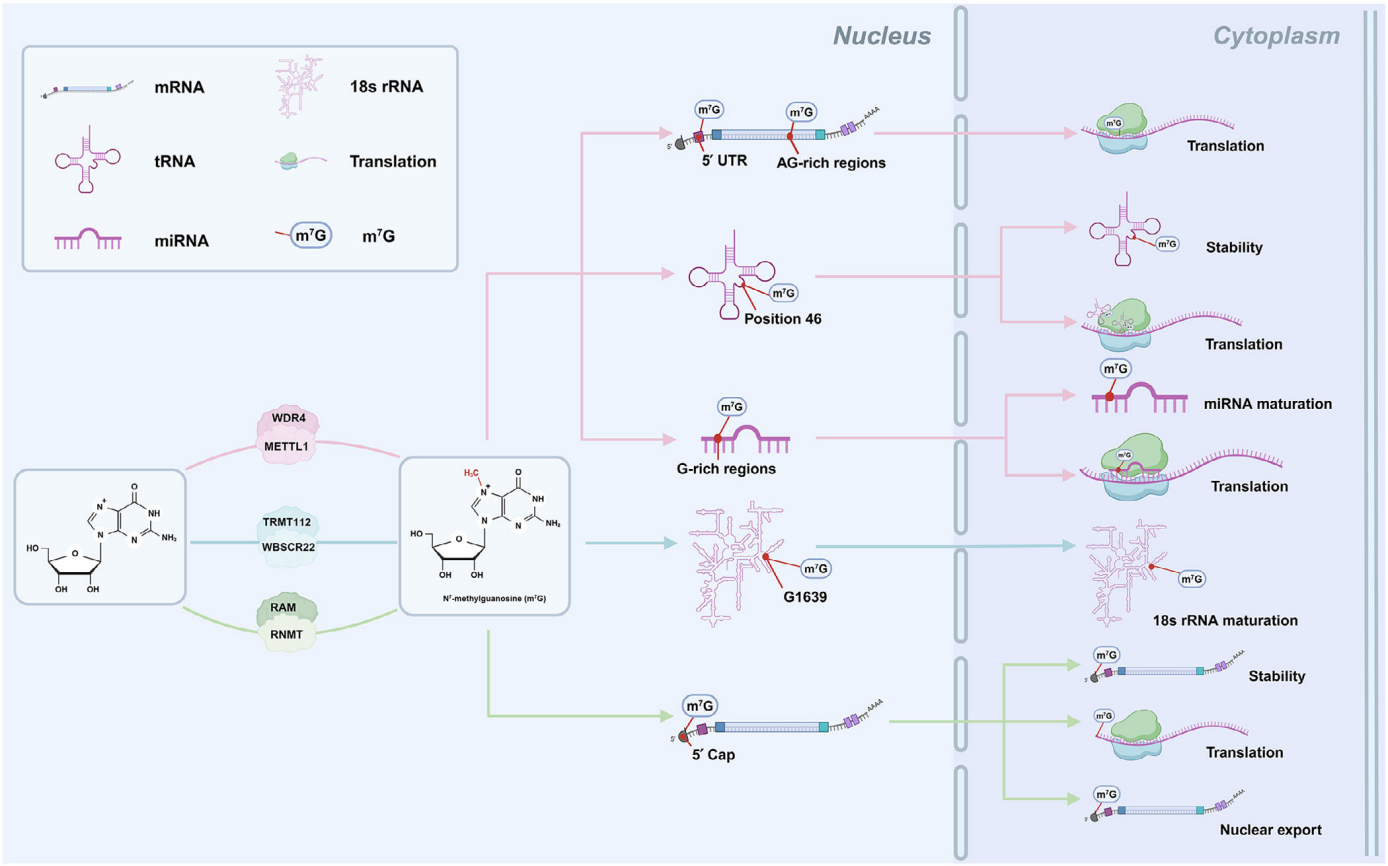
RNA methyltransferase‐mediated diverse m^7^G sites and bio‐effects. The m^7^G methyltransferase complexes are METTL1/WDR4, WBSCR22/TMRT112 and RNMT/RAM, which insert the m^7^G modifications into target RNA molecules, including mRNA, tRNA, rRNA and miRNA. In humans, the m^7^G modification presents in 5′ cap of mRNA, 5′ UTR and AG‐rich regions of internal mRNA, G‐rich regions of miRNA, position 46 of tRNA and G1639 of 18s rRNA. The bio‐effects are mediated by different RNA methyltransferases acting on the RNA stability, maturation, translation and nuclear export.

The m^7^G is the most ubiquitous messenger RNA (mRNA) cap modification that exists in nearly all eukaryotic cells and viral mRNAs and is also present in transfer RNA (tRNA), ribosomal RNA (rRNA) and microRNA (miRNA).^15^, ^16^, ^17^, ^18^, ^19^ m^7^G modification plays critical roles in different biological processes and is mediated by corresponding enzymes (Table 1 and Figure 1). METTL1 and WD repeat domain 4 (WDR4) are the most significant components of the methyltransferase complex, and METTL1 is primarily responsible for mediating m^7^G methylation, whereas WDR4 may help stabilise the heterodimer complex and RNA binding.^20^, ^21^ METTL1/WDR4 catalyses m^7^G at position 46 of tRNA, stabilising the tRNA structure and protecting it from decay through making triple‐base interactions with C13 and G22.^13^, ^22^ Depletion of METTL1 or WDR4 reduces m^7^G tRNA levels and decreases the expression of genes associated with a wide range of biological functions. In addition, METTL1/WDR4 is the writer of the high m^7^G modifications at internal human mRNA sites, including the 5′ untranslated region (UTR) and AG‐rich contexts, which increase the translation efficiency of mRNAs.^18^, ^19^, ^23^ Moreover, METTL1/WDR4 mediates m^7^G modification in miRNAs at G‐rich regions, thereby methylating the primary miRNA (pri‐miRNA) transcript to accelerate its processing to precursor miRNA and maintaining the high maturity levels of pri‐miRNAs by destabilising G‐quadruplexes.^24^ The methyltransferase Williams–Beuren syndrome chromosome region 22 (WBSCR22) partners with its metabolic stabiliser tRNA methyltransferase activator subunit 11–2 (TRMT112) and plays important roles in the biogenesis of small ribosomal subunits in human cells. The WBSCR22/TRMT112 complex facilitates the maturation of 18S rRNA by mediating m^7^G methylation at a specific G1639 location.^25^ The RNA cap methyltransferase in mammals is known as RNA guanine‐7 methyltransferase (RNMT), which has a relatively weak affinity for RNA as a monomer. RNMT‐activating miniproteins (RAMs) can stabilise the RNMT structure and ensure optimal positioning of the RNMT lobe and its adjacent a‐helix hinge in the active sites, thereby promoting substrate binding.^26^, ^27^ Previous research has shown that the RNMT/RAM complex catalyses the formation of an N^7^‐methylated guanosine cap structure to stabilise mRNAs and plays an essential role in their export from the nucleus and translation in the cytoplasm.^28^, ^29^ In summary, m^7^G modification is a potent epigenetic modification that regulates various biological processes. When one key enzyme becomes dysfunctional, this dynamic modification is disrupted, resulting in human diseases.

**TABLE 1:** m^7^G modification‐related factors.

Factors	Intracellular localisation	Target RNA	m^7^G sites	Functions	References
METTL1/WDR4	Nucleus and cytoplasm	tRNA	Position 46	Increased tRNA stability and regulated mRNA translation	^13^, ^22^
		mRNA	5′ UTR and AG‐rich regions	Regulated mRNA translation	^18^, ^19^, ^23^
		miRNA	G‐rich regions	Maintained miRNA maturity and regulated mRNA translation	^24^
WBSCR22/TRMT112	Nucleus	18s rRNA	G1639	Promoted 18s rRNA maturation	^25^
RNMT/RAM	Nucleus and cytoplasm	mRNA	5′ cap	Increased mRNA stability, regulated mRNA translation and nuclear export	^26^, ^27^, ^28^, ^29^

## 3 OVERVIEW OF METTL1

### 3.1 Characteristics of METTL1

**FIGURE 2:**
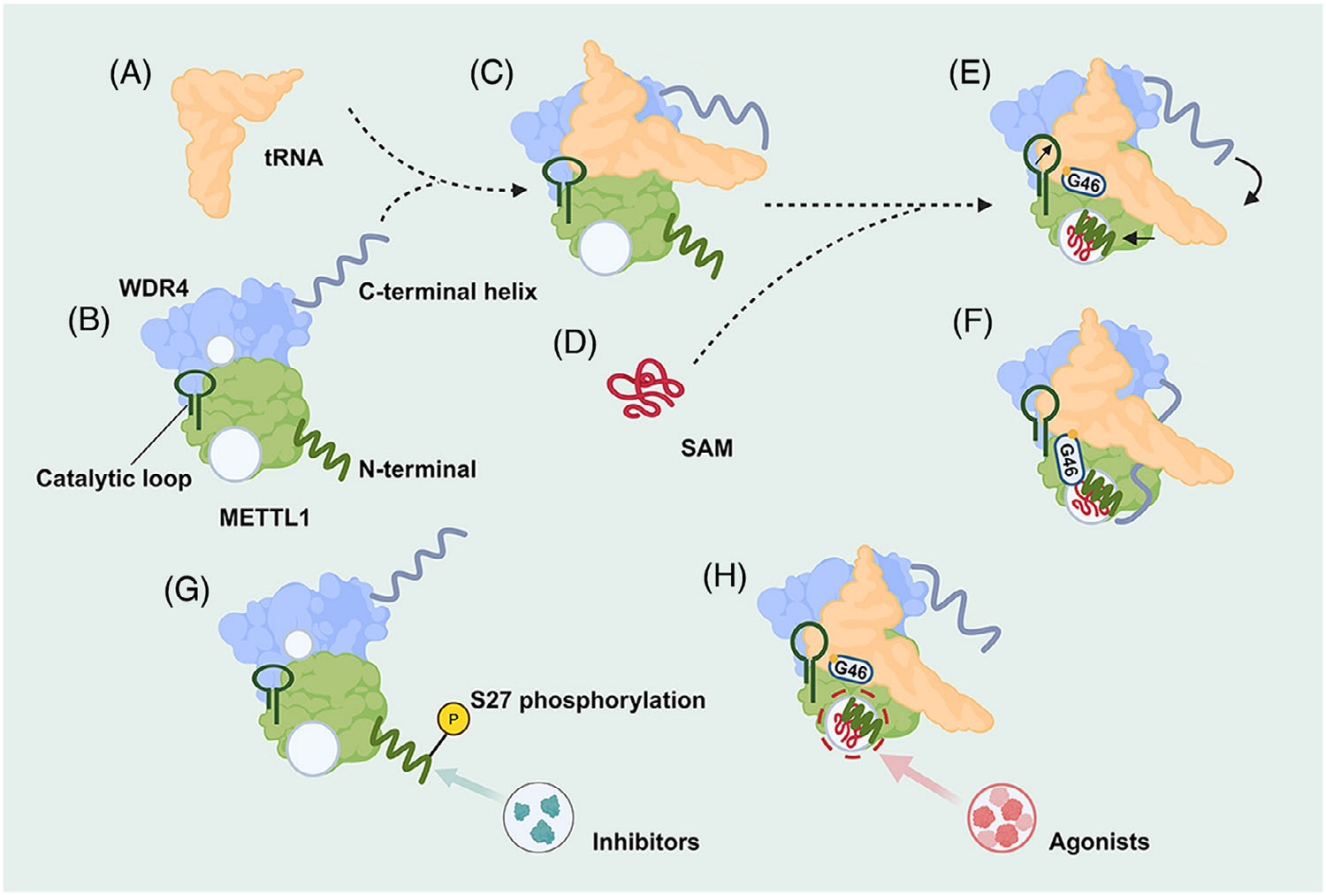
Mechanistic model for tRNA m^7^G methylation by METTL1–WDR4. (A and B) The stable heterodimeric METTL1–WDR4 protein complex provides a docking site for the tRNA elbow. (C and D) The protein‐RNA docking can occur without cofactor, and METTL1 can bind SAM without tRNA. (E and F) When both tRNA and SAM are bound, METTL1 N‐term becomes ordered sandwiched between RNA and SAM, and WDR4 C‐term attaches to the METTL1 N‐term to stabilise the bound RNA together. (G) The inhibitors or S27 phosphorylation of the N‐terminal region of METTL1 can effectively reduce methyltransferase activity by locally disrupting the catalytic centre. (H) The agonists interact with SAM in close proximity to the active centre of METTL1, potentially lowering the energy barrier of the substrate RNA methylation reactions.

The m^7^G at tRNA position 46 (m^7^G46) is one of the most conserved modifications from bacteria to humans that modulates steady‐state tRNA levels to affect cell growth.^13^, ^30^ METTL1 is a mammalian m^7^G writer, and its mechanism of catalysing tRNA modification is critical for understanding how m^7^G contributes to normal physiology and pathophysiology in mammals (Figure 2).

METTL1 alone does not bind tRNA well, but the ternary complex is readily observed in the presence of WDR4. METTL1 and WDR4 bury a total of approximately 1746 Å^2^ of surface area containing many hydrophobic interactions, and polar residues such as R170 and E167 of WDR4 and K143 of METTL1 stabilise the interdomain contacts, which are important for the optimal activity of METTL1–WDR4. Upon METTL1/WDR4 complex formation, the heterodimer forms a composite docking site that identifies the elbow region of tRNAs by shape and charge complementarity. When S‐adenosylmethionine (SAM) is bound, the METTL1 N‐term is ordered between the bound cofactor and the tRNA, while the catalytic loop extends toward the tRNA, strengthening the grip on the outer tRNA elbow by the WDR4 C‐terminal helix rigidifying against the METTL1 N‐term. The same METTL1 N‐term replaces G46 to stack with R24 in the helical core to activate the tRNA, unleashing the guanine to flip out and inserting into the active site containing three acidic residues critical for catalysis and SAM, poised for methylation at the N7 position. Truncations or modifications of the METTL1 N‐term prevent the proper organisation of the catalytic pocket, providing a structural explanation for how METTL1 is regulated.^31^

Recent findings suggest that overexpression of METTL1 is linked to diseases in numerous contexts.^12^, ^14^ Understanding the mechanisms underlying the specific methyltransferase function and regulation of METTL1 provides new insight for future research on the implications of METTL1 in disease.

### 3.2 Biological functions of METTL1

**FIGURE 3:**
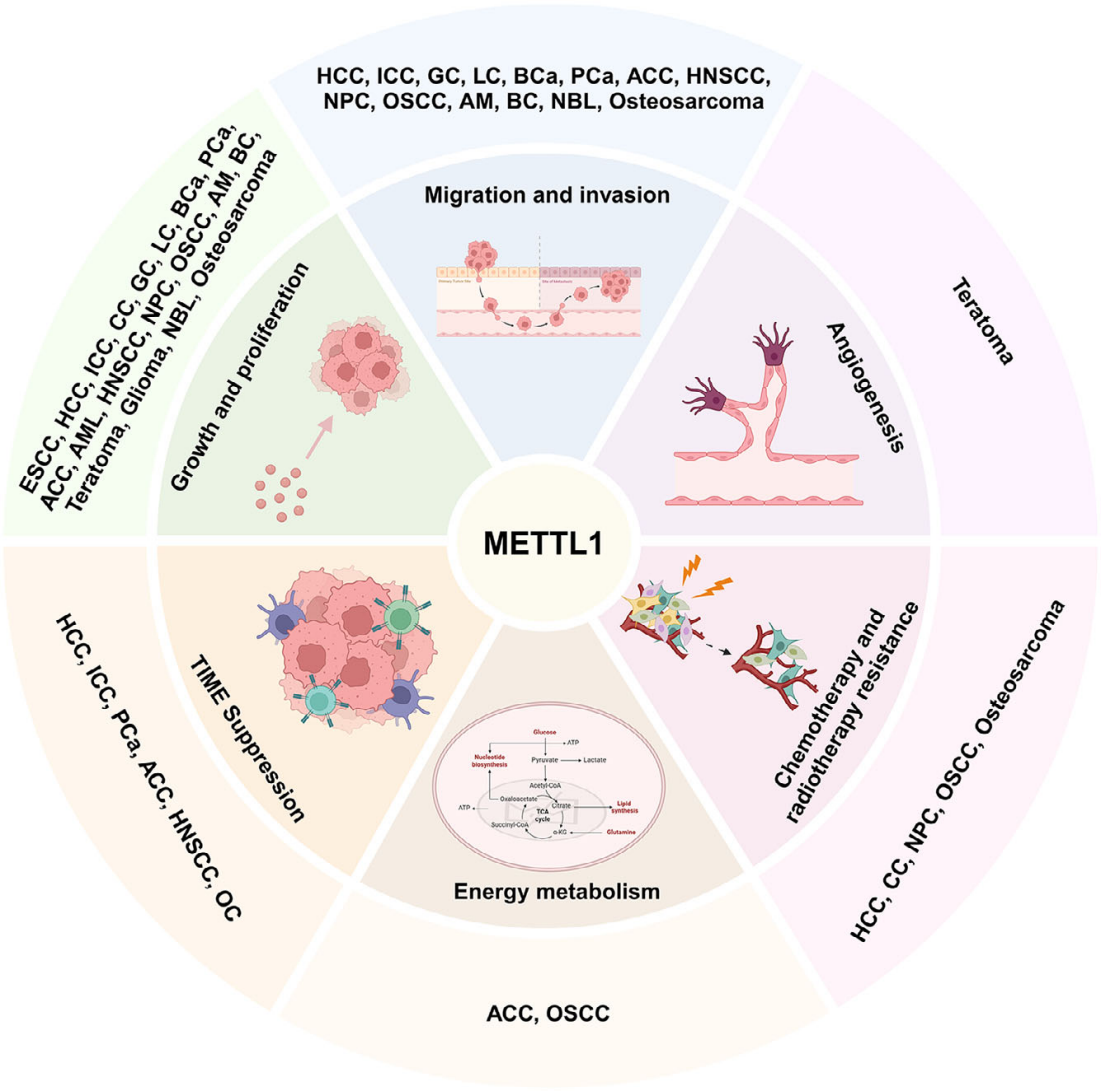
The biological function of METTL1 in cancer. METTL1 regulates the processes involved in the development of cancer, including tumour proliferation, migration, invasion, angiogenesis, chemotherapy and radiotherapy resistance, energy metabolism and TIME suppression.

Recent studies have shown that METTL1 is closely associated with processes involved in the progression of diseases, including tumour proliferation, migration, invasion, angiogenesis, chemo‐ and radiotherapy resistance, energy metabolism and TIME suppression. We present a summary of recent findings concerning the functions of METTL1 in disease (Figure 3).

#### 3.2.1 Cell cycle progression

Cell proliferation is the foundation of growth and development in organisms.^32^, ^33^ However, indefinite proliferation is an important hallmark of cancer. Many studies have shown that METTL1 facilitates cell proliferation in cancer by regulating several different targets or pathways. METTL1 promotes colorectal cancer cell proliferation by attenuating checkpoint kinase 2 (CHEK2)‐induced G1/S phase arrest.^34^ In hepatocellular carcinoma (HCC), METTL1 knockdown suppresses the translation of cyclin A2, leading to the G2/M cell cycle arrest.^21^, ^35^ Furthermore, METTL1 can indirectly regulate the proliferation of castration‐resistant prostate cancer (CRPC) cells through cyclin‐dependent kinase 14 (CDK14).^36^ Moreover, the METTL1–activating transcription factor 5 (ATF5)–INCA1 axis inhibited cardiomyocyte proliferation by blocking the interaction of CDK2 with cyclin A1, resulting in S to G2/M cell cycle arrest.^37^ In addition, METTL1 inhibited breast cancer (BC) cell cycle progression and proliferation by promoting the translation of growth arrest and DNA damage 45 alpha (GADD45A) and retinoblastoma protein 1 (RB1) mRNAs, selectively blocking the G2/M phase of the cell cycle.^38^ In summary, these observations reveal that METTL1 is essential for the proliferation of cancer cells.

#### 3.2.2 Migration and invasion

More than 90% of cancer‐related fatalities from solid tumours are caused by migration and invasion.^39^ METTL1 knockdown attenuates HCC cell migration and invasion, whereas METTL1 overexpression results in the opposite effects.^35^, ^40^ Studies have revealed that METTL1 overexpression enhances the migrative and invasive abilities of osteosarcoma and adrenocortical carcinoma (ACC) cells.^41^, ^42^ In addition, the migration and invasion capacities of lung cancer (LC) cells are also enhanced by METTL1 overexpression, and the same is true for bladder cancer (BCa), nasopharyngeal carcinoma (NPC) and oral squamous cell carcinoma (OSCC).^43^, ^44^, ^45^, ^46^, ^47^ In conclusion, substantial evidence corroborates that METTL1 may serve as a driving force in cancer metastasis.

#### 3.2.3 Angiogenesis

Angiogenesis plays a vital role in the development process of organisms, contributing significantly to tissue repair, growth and the maintenance of normal physiological functions.^48^ Moreover, angiogenesis is a fundamental process of tumour growth and metastasis that can provide nutrition for tumour tissue metabolism.^49^ METTL1 silencing substantially accelerated human induced pluripotent stem cell (hiPSC) differentiation toward a mesoderm fate while suppressing neuroectoderm differentiation, promoting angiogenesis and enhancing teratoma formation.^50^ Moreover, hiPSC‐derived endothelial progenitor cells with METTL1 knockdown robustly induced vascular smooth muscle cell proliferation in a coculture system.^51^ In addition, METTL1 facilitates postischemic angiogenesis by promoting vascular endothelial growth factor A (VEGFA) mRNA translation in a m^7^G methylation‐dependent manner.^52^ However, further research is needed to determine whether METTL1 is involved in the angiogenesis of other malignancies and affects the malignant process of tumours.

#### 3.2.4 Chemotherapy and radiotherapy resistance

Chemotherapy and radiotherapy are both widely used to treat solid tumours; however, resistance to chemotherapy and radiotherapy limits therapeutic efficacy.^53^ METTL1 enhances anlotinib resistance in OSCC by increasing the translation of enzymes associated with the respiratory chain and increasing oxidative phosphorylation (OXPHOS) capacity.^46^ A recent study revealed that METTL1 increases osteosarcoma chemoresistance to doxorubicin by altering oncogenic mRNA translation.^42^ However, METTL1/pri‐miR‐26a/ferritin heavy chain (FTH1) axis signalling enhances ferroptosis in osteosarcoma and strengthens the sensitivity of osteosarcoma cells to chemotherapeutic drugs.^54^ Furthermore, METTL1 not only enhances the translation of epidermal growth factor receptor (EGFR) pathway genes to trigger lenvatinib resistance in HCC but also promotes DNA double‐strand break (DSB) repair and renders HCC cells resistant to ionising radiation (IR).^55^ These findings indicate that METTL1 is involved in chemoradiotherapy resistance in cancer, suggesting that METTL1 could be a potential target for reversing chemoradiotherapy resistance.

#### 3.2.5 Energy metabolism

One of the most common symptoms of disease is abnormal energy metabolism.^33^, ^56^ Experimental assays revealed that METTL1 is associated with active glycolysis metabolism by favourably influencing the expression of the glycolysis rate‐limiting enzyme HK1 in ACC.^41^ In addition, METTL1 drives a metabolic shift from glycolysis to OXPHOS, with METTL1 knockdown strikingly reducing ROS generation and mitochondrial OXPHOS complex expression levels in anlotinib‐resistant OSCC cells.^46^ Collectively, these data suggest that METTL1 may function as an oncogene in cancer by regulating glycolysis and mitochondrial metabolism.

#### 3.2.6 TIME suppression

Elements within the TIME coordinate tumour immunity, and the TIME significantly influences tumour biological behaviour.^57^, ^58^ METTL1 up‐regulates the expression of the chemokines C‐X‐C motif chemokine ligand 5 (CXCL5) and CXCL8 in a m^7^G‐dependent manner, leading to myeloid‐derived suppressor cell (MDSC) accumulation and immunosuppression in HCC and intrahepatic cholangiocarcinoma (ICC).^59^, ^60^ Moreover, high METTL1 expression is associated with decreased cytotoxic CD8^+^ T‐cell infiltration and is positively correlated with the abundance of M2 macrophages.^41^, ^61^ METTL1 also plays a role in the polarisation of tumour‐associated macrophages (TAMs). Elevated METTL1 expression is correlated with increased infiltration of M2‐like macrophages, whereas inhibition of METTL1 induces TAMs toward an M1‐like endotype in preclinical models of prostate cancer (PCa).^61^ These results support the indispensable role of METTL1 in influencing the infiltration of immune cells within tumours and ultimately shaping the immunosuppressive microenvironment.

### 3.3 Regulation of METTL1 expression

**FIGURE 4:**
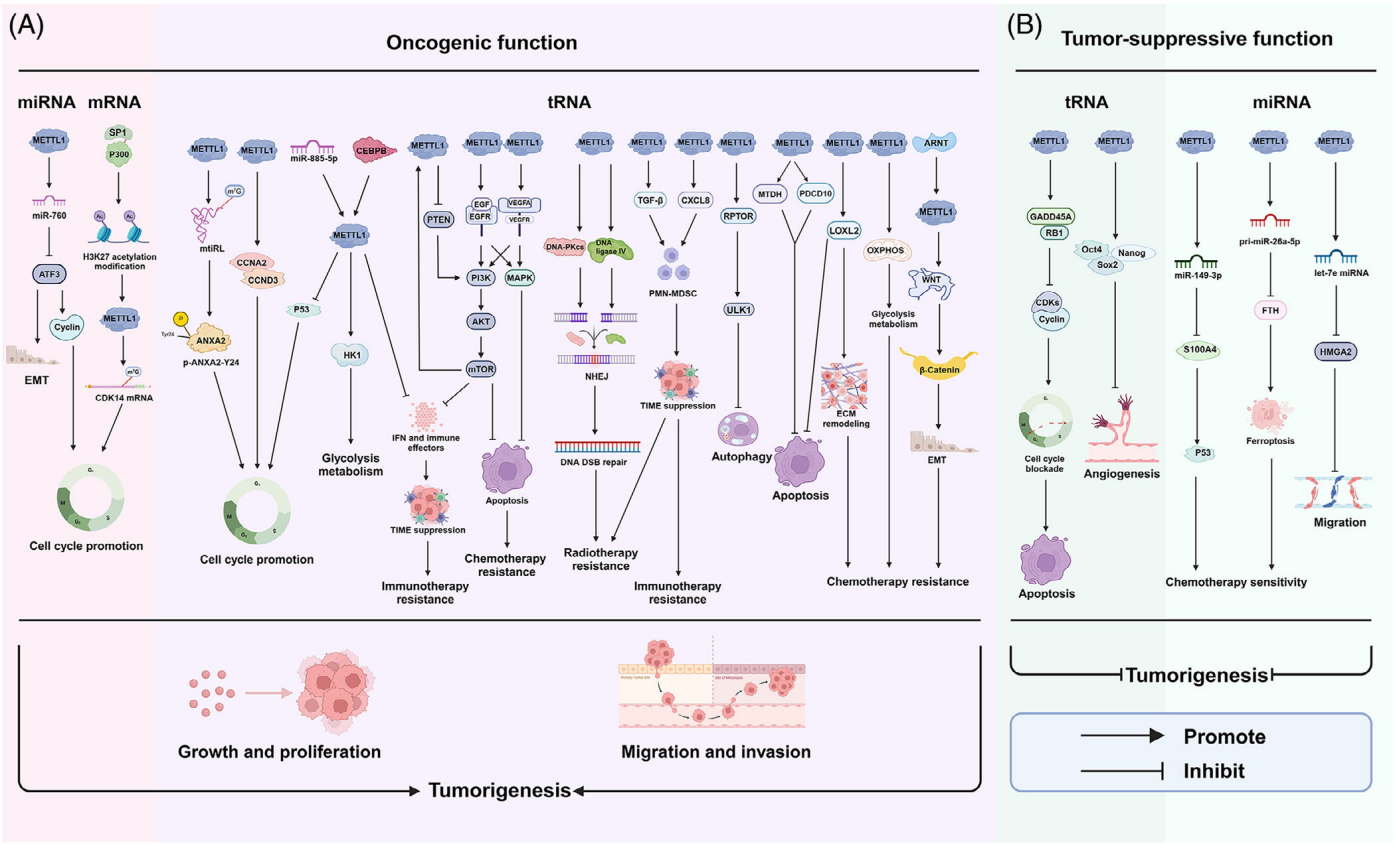
Molecular mechanisms linking regulation of METTL1 to tumourigenesis. METTL1‐mediated m^7^G modification plays an important role as biomarkers and therapeutic targets for tumours and occurs in different RNAs, including tRNA, mRNA and miRNA. (A) Molecular mechanisms of METTL1‐mediated m^7^G modification in promoting tumourigenesis by regulating cancer progression, including tumour proliferation, migration, invasion, autophagy, chemotherapy and radiotherapy resistance, glycolysis metabolism and TIME suppression. (B) Molecular mechanisms of METTL1‐mediated m^7^G modification in inhibiting tumourigenesis by regulating cancer progression, including tumour proliferation, migration, invasion, angiogenesis and chemotherapy resistance.

The majority of research has focused on the function of METTL1 in disease, with only a few studies investigating the causes of aberrant METTL1 expression (Figure 4). According to a recent study, Ah receptor nuclear translocator protein (ARNT) is an upstream transcription factor that negatively regulates METTL1 expression by inhibiting its transcription, hence antagonising the oncogenic role of METTL1 in NPC.^47^ Furthermore, METTL1 is a downstream effector of the phosphatidylinositol‐3‐kinase/protein kinase B/mammalian target of rapamycin (PI3K/AKT/mTOR) pathway and its activation induces elevated expression of METTL1 in PCa.^61^ Data mining of public databases revealed that miR‐885‐5p and CCAAT/enhancer binding protein beta (CEBPB) are upstream regulators of METTL1 involved in ACC progression.^41^ YY1 has been reported to act as a transcriptional factor, activating METTL1 expression during cardiac hypertrophy.^62^ In addition, P300 can form a complex with SP1 and bind to the promoter region of the METTL1 gene via SP1, resulting in the H3K27 acetylation modification of METTL1 and its up‐regulation in CRPC.^36^ Studying the upstream regulatory mechanism of METTL1 is beneficial for us to better understand its biological function in diseases.

### 3.4 Downstream targets of METTL1

The earliest reports indicated that METTL1 can exert oncogenic effects in HCC via the suppression of its downstream target PTEN.^40^ Recent research has revealed that METTL1–m^7^G tRNA modification plays a critical role in promoting ICC cell survival and progression by selectively regulating the translation of oncogenic transcripts, including cell cycle‐related mRNAs and the EGFR and VEGFA pathway genes.^63^ Similarly, the enhancement of EGFR and VEGFA translation in lenvatinib‐resistant hepatocellular cancer has been linked to METTL1 activity.^55^ In recurrent HCC after radiofrequency ablation (RFA), elevated METTL1 expression facilitates the translation of transforming growth factor beta receptor‐2 (TGF‐β2), inducing polymorphonuclear MDSC (PMN‐MDSC) accumulation and decreasing CD8^+^ T‐cell infiltration, resulting in an immunosuppressive tumour microenvironment.^60^ Additionally, METTL1 induces PMN‐MDSC accumulation by enhancing the translation of CXCL8, which promotes anti‐programmed cell death protein‐1 (PD‐1) tolerance in ICC cells and accelerates ICC progression.^59^ Moreover, overexpression of the METTL1 downstream target cyclin D3 (CCND3) may partially rescue the growth and invasion capacity of METTL1‐depleted LC cells.^43^

METTL1 stimulates osteosarcoma malignancy by augmenting mRNA translation of lysyl oxidase‐like 2 (LOXL2), a key downstream target of METTL1 that is required for its function in osteosarcoma.^42^ Interestingly, another study revealed that METTL1 reduces the FTH1 protein level by targeting pri‐miR‐26a and FTH1, evoking ferroptosis and promoting the sensitivity of osteosarcoma cells to chemotherapeutic treatments.^54^ Furthermore, METTL1 overexpression enhances the cytotoxic effects of high‐dose cisplatin on cisplatin‐resistant colon cancer (CR‐CC) cells by positively regulating miR‐149‐3p.^64^

In systemic lupus erythematosus (SLE), lysosomal‐associated membrane protein 3, the CD83 molecule (CD83) and programmed cell death 1 ligand 2 may be downstream targets of METTL1 and are involved in abnormal immunological responses.^65^ Another study revealed that METTL1 increases serine/arginine‐rich splicing factor 9 (SRSF9) expression in a m^7^G‐dependent manner, facilitating alternative splicing and the stabilisation of nuclear factor of activated T cells, cytoplasmic, calcineurin‐dependent 4 (NFATc4) and thus promoting cardiac hypertrophy.^62^ In addition, ATF5 has been identified as a downstream target of METTL1 in the regulation of cardiomyocyte proliferation and cardiac regeneration.^37^

## 4 ROLE OF METTL1 IN VARIOUS DISEASES

### 4.1 The role of METTL1 in tumours

**FIGURE 5:**
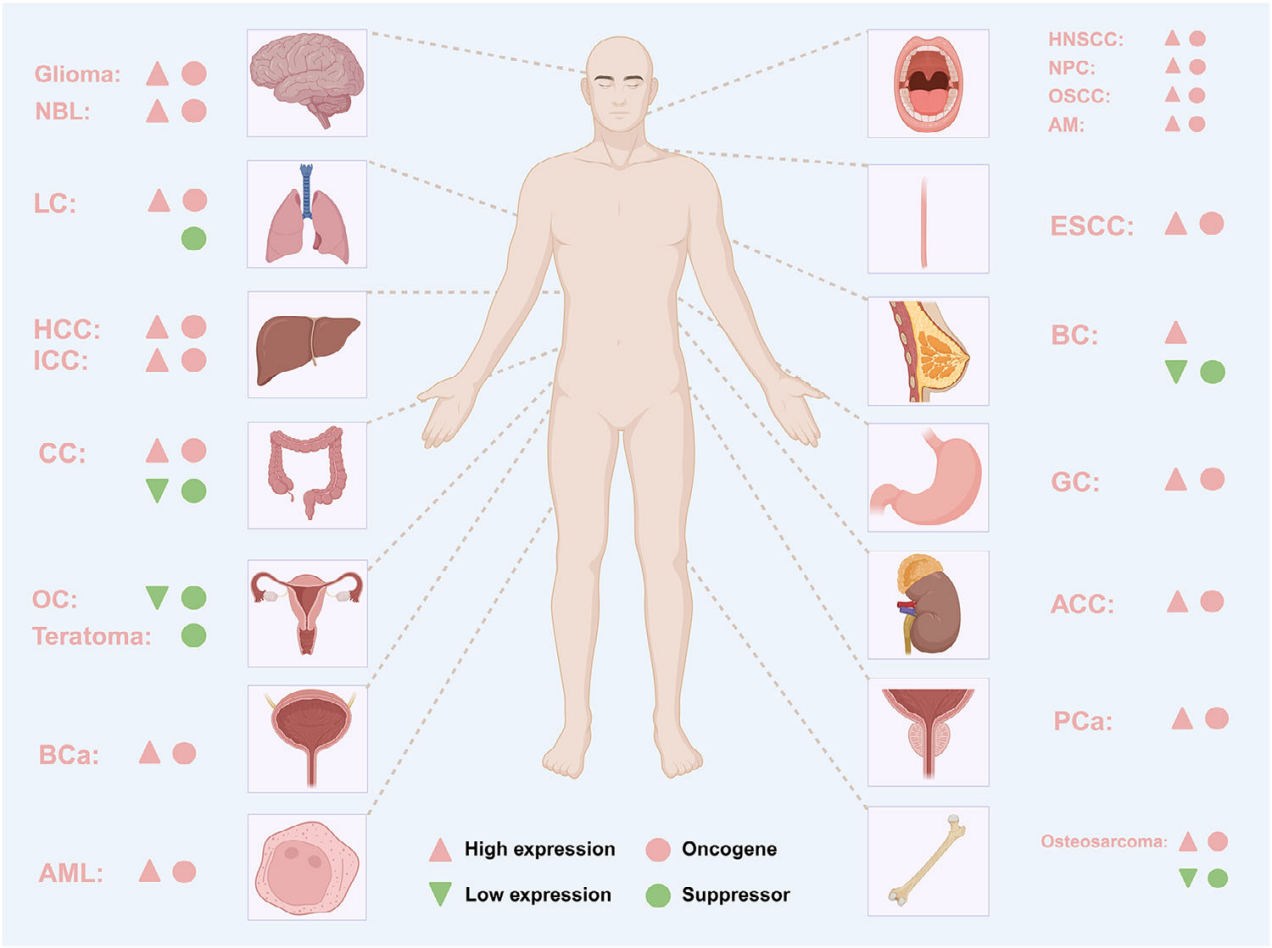
The role of METTL1 in human cancer. Aberrant expression of METTL1 in various human malignancies and its dual roles in the promotion or suppression of tumour progression. ESCC, oesophageal squamous cell carcinoma; HCC, hepatocellular carcinoma; ICC, intrahepatic cholangiocarcinoma; CC, colon cancer; GC, gastric cancer; LC, lung cancer; BCa, bladder cancer; PCa, prostate cancer; ACC, adrenocortical carcinoma; AML, acute myeloid leukaemia; HNSCC, head and neck squamous cell carcinoma; NPC, nasopharyngeal carcinoma; OSCC, oral squamous cell carcinoma; AM, ameloblastoma; BC, breast cancer; OC, ovarian cancer; NBL, neuroblastoma.

Accumulating evidence in recent years has demonstrated that METTL1 plays critical roles in cancer as a m^7^G methyltransferase, either as an oncogene or a tumour suppressor, as summarised in Table 2 and Figure 5.

**TABLE 2:** The functional roles of METTL1 in various types of human cancers.

Cancer type	Cell lines	Roles	m^7^G target	Functions	Mechanism	References
ESCC	KYSE150, KYSE30	Oncogene	tRNA	Growth↑ Proliferation↑	↑METTL1/↑RPTOR/ ↑P‐ULK1/↓autophagy	^68^
HCC	Huh7, MHCC97H	Oncogene	tRNA	Proliferation↑ Migration↑ Invasion↑ Apoptosis↓	↑METTL1/↑EGF/EGFR and VEGFA/VEGFR1/↑p‐AKT and p‐p44/42 MAPK	^35^
	HepG2, Huh7	Oncogene	tRNA	Proliferation↑ Migration↑	↑METTL1/↓PTEN	^40^
	MHCC97H, SNU449	Oncogene	tRNA	Radiotherapy resistance↑ DNA DSB repair↑	↑METTL1/↑DNA‐PKcs and DNA ligase IV/↑NHEJ	^70^
	MHCC97H, SNU449	Oncogene	tRNA	Radiotherapy resistance↑ TIME↓	↑METTL1/↑TGF‐β/↑PMN‐MDSC	^60^
	Huh7, PLC/PRF/5	Oncogene	tRNA	Chemotherapy resistance↑ Proliferation↑ Apoptosis↓	↑METTL1/↑EGFR	^55^
ICC	HuCCT1, RBE	Oncogene	tRNA	Growth↑ Migration↑ Invasion↑ Apoptosis↓	↑METTL1/↑CCNA2 and EGFR	^63^
	HuCCT1, RBE	Oncogene	tRNA	Growth↑ Proliferation↑ TIME↓ Immunotherapy resistance↑	↑METTL1/↑CXCL8/↑PMN‐MDSC	^59^
CC	HCT116, SW480, SW620	Suppressor	miRNA	Chemotherapy resistance↓	↑METTL1/↑miR‐149‐3p/↓S100A4/↑p53	^64^
	HCT116, RKO	Oncogene	No study	Growth↑ Proliferation↑	↑METTL1/↓CHEK2	^34^
GC	AGS, HGC‐27	Oncogene	No study	Proliferation↑ Migration↑ Invasion↑ Apoptosis↓	↑TSPAN31/↑METTL1	^76^
LC	A549, NCI‐H1299	Oncogene	tRNA	Growth↑ Proliferation↑ Migration↑ Invasion↑	↑METTL1/↑CCND3	^43^
	A549	Suppressor	miRNA	Migration↓	↑METTL1/↑let‐7e miRNA/↓HMGA2	^24^
BCa	T24, UMUC‐3	Oncogene	tRNA	Proliferation↑ Migration↑ Invasion↑	↑METTL1/↑EGFR/EFEMP1–PI3K–AKT	^44^
	T24, UMUC‐3	Oncogene	miRNA	Proliferation↑ Migration↑	↑METTL1/↑miR‐760/↓ATF3	^45^
	T24, SV‐HUC‐1	Oncogene	tRNA	Proliferation↑ Migration↑	↑METTL1/↑mtiRL / ↑p‐ANXA2‐Y24	^85^
PCa	PC3 DU145, 22Rv1	Oncogene	tRNA	Growth↑ Proliferation↑ Apoptosis↓ TIME↓ Immunotherapy resistance↑	↓PTEN/↑PI3K–AKT–mTOR/↑METTL1/↓5′tRFs/↓IFN and immune effectors	^61^
	LNCaP‐AI, C4‐2	Oncogene	mRNA	Proliferation↑ Invasion↑	↑P300‐SP1/↑H3K27ac/↑METTL1/↑CDK14	^36^
ACC	H295R, SW13	Oncogene	tRNA	Growth↑ Proliferation↑ Migration↑ Invasion↑ Glycolysis metabolism↑ TIME↓ Immunotherapy resistance↑	↑miR‐885‐5p and CEBPB/↑METTL1/↑HK1, ↓P53 and IFN	^41^
AML	MOLM‐13	Oncogene	tRNA	Growth↑ Proliferation↑	↑METTL1/↑Arg‐TCT‐4‐1 tRNA/↑AGA	^89^
HNSCC	SCC9, SCC15	Oncogene	tRNA	Proliferation↑ Migration↑ Invasion↑ Apoptosis↓ TIME↓	↑METTL1/↑PI3K–AKT–mTOR	^90^
NPC	5‐8F, 6–10B	Oncogene	tRNA	Growth↑ Proliferation↑ Migration↑ Invasion↑ Apoptosis↓ EMT↑ Chemotherapy resistance↑	↓ARNT/↑METTL1/↑WNT/β‐Catenin/↑c‐Myc and Cyclin D1	^47^
OSCC	SCC9, SCC15	Oncogene	tRNA	Growth↑ Proliferation↑ Migration↑ Apoptosis↓ Glycolysis metabolism↑ Chemotherapy resistance↑	↑METTL1/↑OXPHOS	^46^
AM	hTERT‐AM	Oncogene	tRNA	Growth↑ Proliferation↑ Migration↑ Invasion↑	↑METTL1/↑MAPK	^96^
BC	MCF‐7	Oncogene	tRNA	Growth↑ Proliferation↑ Migration↑ TIME↓	↑METTL1/↑co‐expressed genes	^99^
	MDA‐MB‐231, MCF‐7	Suppressor	tRNA	Growth↓ Proliferation↓ Migration↓ Invasion↓ Apoptosis↑	↑METTL1/↑GADD45A and RB1/↓CDKs and CCNB1	^38^
OC	OC cells	Suppressor	No study	TIME↑	↑METTL1/↑immune effectors	^100^
Teratoma	HiPSCs	Suppressor	tRNA	Proliferation↑ Self‐renewal↑ Differentiation↓ Angiogenesis↓	↑METTL1/↑Oct4, Nanog and Sox2	^50^
Glioma	U87	Oncogene	tRNA	Proliferation↑	↑METTL1/↑MAPK	^103^
NBL	KELLY, BE2C	Oncogene	tRNA	Proliferation↑ Migration↑ Apoptosis↓	↑METTL1/↑MTDH and PDCD10	^105^
Osteosarcoma	HOS, 143B	Oncogene	tRNA	Growth↑ Proliferation↑ Migration↑ Apoptosis↓ Chemotherapy resistance↑	↑METTL1/↑LOXL2	^42^
	U2OS, 143B	Suppressor	miRNA	Proliferation↓ Migration↓ Invasion↓ Ferroptosis↑ Chemotherapy resistance↓	↑METTL1/↑pri‐miR‐26a‐5p/↓FTH1	^54^

#### 4.1.1 Gastrointestinal tumours

##### Oesophageal squamous cell carcinoma

Oesophageal cancer is an aggressive malignancy and the eighth most common cause of cancer death, accounting for more than 70% of all oesophageal cancer cases.^66^ The 5‐year survival rate of oesophageal squamous cell carcinoma (ESCC) patients remains very low since it is related to extensive lymphatic spread and vascular invasion; hence, identifying effective therapeutic targets for better treatment of ESCC is highly important.^67^ One study revealed that METTL1 and WDR4 are notably up‐regulated in ESCC tissues and are associated with poor prognosis. Mechanistically, abnormal activation of the mTOR pathway results in the phosphorylation and activation of ULK1, mediating the negative regulation of autophagy and initiating the malignant proliferation of ESCC cells. By using tRNA reduction and cleavage sequencing (TRAC‐seq) and polyribosome‐seq, Han et al.^68^ revealed that METTL1 knockdown leads to decreased expression of m^7^G‐modified tRNAs and reduces translation of a subset of oncogenic transcripts enriched in regulatory associated protein of mTOR complex 1 (RPTOR)/ULK1/autophagy signalling. According to these studies, METTL1 and its downstream signalling axis could be the promising therapeutic targets for ESCC treatment.

##### Hepatocellular carcinoma

HCC accounts for the majority of primary liver malignancies (80–90%) and is the fifth most common cause of cancer‐related death.^66^ A recent study revealed that the levels of METTL1 and WDR4 are elevated in HCC and are associated with advanced tumour stages and poor patient survival. METTL1 knockdown decreases the mRNA translation of EGF/EGFR and VEGFA/VEGFR1, reduces the activities of the corresponding downstream signalling pathways, as well as those of AKT and MAPK, and therefore inhibits proliferation and metastasis in HCC.^35^ Additionally, METTL1 enhances the growth and migration of HCC cells via the suppression of PTEN signalling, suggesting that METTL1 is a promising target for HCC treatment.^40^

Radiotherapy is an increasingly essential therapeutic strategy that plays pivotal roles in the management of HCC; however, resistance to radiotherapy is a primary obstacle to successful treatment outcomes.^69^ Previous investigations have demonstrated that elevated METTL1 expression in tumour tissues is strongly associated with poor prognostic outcomes in HCC patients undergoing radiotherapy. METTL1‐mediated m^7^G tRNA modification selectively regulates the translation of DNA‐dependent protein kinase catalytic subunit (DNA‐PKcs) or DNA ligase IV with increased frequencies of m^7^G ‐related codons after IR treatment, thereby resulting in increased nonhomologous end‐joining (NHEJ)‐mediated DNA DSB repair efficiency and rendering HCC cells resistant to IR.^70^ Furthermore, disrupting METTL1/TGF‐β/PMN‐MDSC axis significantly mitigates tumour progression by decreasing PMN‐MDSC accumulation and restoring the CD8^+^ T‐cell population after RFA.^60^ Thus, METTL1 might be a reliable biotarget to increase the efficacy of radiotherapy in HCC and prevent HCC recurrence after RFA treatment.

The tyrosine kinase inhibitor (TKI) lenvatinib is a first‐line treatment for advanced HCC, but resistance still frequently occurs in a considerable proportion of patients.^71^ One study revealed that METTL1 and WDR4 are dramatically up‐regulated in lenvatinib‐resistant cells. Following lenvatinib treatment, METTL1 knockdown overrides resistance by impairing the proliferation capacity and promoting the apoptosis of HCC cells. In addition, METTL1 overexpression induces lenvatinib resistance by increasing the translation of EGFR pathway genes.^55^ These findings indicate that up‐regulation of METTL1 strengthens lenvatinib resistance in HCC and confers sensitivity to METTL1 targeting, providing a promising strategy to overcome resistance. These studies have revealed new insights into the role of METTL1 in HCC and provide a molecular basis for HCC diagnosis and treatment.

##### Intrahepatic cholangiocarcinoma

ICC is a severe malignant tumour that currently comprises 10–20% of primary hepatic tumours.^72^ One study revealed that METTL1 and WDR4 are strikingly increased in ICC and are associated with poor prognosis. This study further revealed the critical role of METTL1‐mediated m^7^G tRNA modification in accelerating ICC cell survival and progression by selectively regulating the translation of oncogenic transcripts, including the cell‐cycle‐related mRNAs cyclin A2 (CCNA2), cyclin D2 (CCND2), CDK6 and CDK8, as well as EGFR pathway genes.^63^ Another study demonstrated that PMN‐MDSCs are enriched in advanced ICC cells and strongly correlated with METTL1. The key translational targets of METTL1 are CXCL8 in humans and CXCL5 in mice, which can facilitate its crucial immunomodulator function in promoting PMN‐MDSC accumulation in the TIME and ICC progression. In preclinical ICC mouse models, co‐blockade of METTL1 and its downstream chemokine pathway enhances anti‐PD‐1 efficacy.^59^ In conclusion, the mechanisms of METTL1‐mediated changes in the TIME of ICC cells provide new insights for the development of efficient ICC immunotherapeutic strategies.

##### Colon cancer

CC is a common malignant tumour with high morbidity and mortality worldwide, seriously dampening quality of human life.^73^ A study utilising the CR‐CC cells revealed that METTL1 expression was lower in CR‐CC cells than in paired cisplatin‐sensitive CC cells. In addition, the S100 calcium‐binding protein A4 (S100A4)/p53 axis is the downstream target of METTL1 and miR‐149‐3p, and overexpressing either METTL1 or miR‐149‐3p increases p53 protein levels and amplifies the cytotoxic effects of cisplatin in CR‐CC cells, which can be reversed by up‐regulating S100A4. In general, overexpression of METTL1 sensitises CR‐CC cells to cisplatin by modulating the miR‐149‐3p/S100A4/p53 axis.^64^ These studies provide new gene targets for CC treatment. Additionally, another study revealed that the expression of METTL is markedly up‐regulated in colorectal cancer tissues compared with normal tissues and is positively correlated with poor prognosis. METTL1 knockdown suppresses CC cell growth and the G1/S phase transition. Further functional experiments indicated that METTL1 may directly interact with the cell cycle‐related gene CHEK2 and suppress its protein expression, thereby attenuating CHEK2‐induced G1/S phase arrest.^34^ Taken together, these results indicate that METTL1 has the ability to modulate CC cell proliferation and progression in a CHEK2‐dependent manner, suggesting that METTL1 is a potential therapeutic target for CC.

##### Gastric cancer

Gastric cancer (GC) is one of the most common malignant tumours of the human digestive tract and it is usually diagnosed at an advanced stage once it is detected due to ambiguous or nonspecific symptoms.^74^ The overall survival rate of patients with GC is still a cause of concern, and detecting genes that are closely related to GC development and have diagnostic and therapeutic implications is particularly important.^75^ One study revealed that the expression level of METTL1 is greater in GC tissues than in normal tissues. The regulatory ability of METTL1 to promote the proliferation and migration of GC cells while inhibiting cell apoptosis may be mediated by tetraspanin 31 (TSPAN31).^76^ Moreover, this may be related to the fact that METTL1 is a flanking gene of the oncogene CDK4, which is enriched on chromosome 12q14–q15.^77^ Although the mechanism by which METTL1 regulates GC cells has not been thoroughly investigated and should be further explored in future studies, METTL1 could be used as a new target for early detection, diagnosis and treatment of GC.

#### 4.1.2 Respiratory tumours

##### Lung cancer

LC is one of the most common cancers and a leading cause of cancer‐related death worldwide.^78^ Mis‐regulated epigenetic modifications such as DNA methylation, and RNA and histone modifications, play important roles in LC progression.^79^ One study revealed that the expression levels of METTL1 and WDR4 are noticeably elevated in human LC samples and are negatively associated with patient prognosis. The results of ribosome profiling with nucleotide resolution sequencing (RNC‐seq) confirmed that impaired m^7^G tRNA modification upon METTL1/WDR4 depletion could decrease the translation of mRNAs related to the proliferation, invasion and tumourigenic capacities of LC cells. Furthermore, overexpression of the METTL1 downstream target CCND3 partially rescues the growth and invasion capacities of METTL1‐depleted LC cells.^43^ These findings provide strong evidence that METTL1 promotes LC growth and invasion through the regulation of m^7^G tRNA modifications, selectively promoting the translation of oncogenes and modulating the processes of cell cycle‐related mRNAs. However, another study confirmed that METTL1 inhibits the proliferation of LC A549 cells by promoting the processing of pri‐let‐7e miRNA transcripts into precursor pre‐let‐7e miRNA and mature let‐7e miRNA, which negatively regulates the oncogene high mobility group AT‐hook 2 (HMGA2).^24^ HMGA2 is known to facilitate the epithelial–mesenchymal transition (EMT) and expedite cancer progression, thereby contributing to unfavourable patient outcomes.^80^ METTL1 knockdown increases HMGA2 mRNA expression and protein levels in LC cells, whereas the transfection of mature let‐7e miRNA reverts the up‐regulation of the HMGA2 protein caused by METTL1 depletion.^24^ Therefore, the functions of METTL1 in LC are still not fully understood, and further in‐depth research is essential for the development of new therapeutic strategies for effective LC treatment.

#### 4.1.3 Urological tumours

##### Bladder cancer

BCa is the fifth most common malignancy and frequently exhibits rapid progression, distant metastasis and a consequently poor prognosis.^66^, ^81^, ^82^ Thus, it is imperative to identify potential therapeutic targets for improved BC treatment. A study confirmed that METTL1‐mediated m^7^G tRNA modification promoted the proliferation, migration and invasion of BCa cells by increasing the protein expression of EGFR/EGF‐containing fibulin‐like extracellular matrix protein 1 (EFEMP1).^44^ EGFR is a key receptor involved in signalling pathways that drive tumour progression. The PI3K/AKT pathway, which is downstream of EGFR, can be activated by the EGFR–ligand interaction, driving cancer cell proliferation, survival and invasion.^83^ Furthermore, the binding EGFR results in the autophosphorylation of EGFR and the activation of downstream signalling pathways that accelerate BCa progression. In addition, Xie et al.^45^ reported that METTL1 drives tumour progression in BCa by promoting the processing of miR‐760 in a m^7^G‐dependent manner through the combination of miRNA‐seq and m^7^G methylated RNA immunoprecipitation sequencing (MeRIP‐seq). ATF3 is a potential target of miR‐760 and can be degraded by miR‐760. ATF3 overexpression significantly represses the proliferation and migration of BCa cells by influencing the expression of cell cycle‐associated proteins and EMT‐associated proteins.^84^ Taken together, these findings reveal a regulatory axis composed of METTL1/miR‐760/ATF3 in regulating BCa progression and provide a new avenue for BCa treatment.

Surprisingly, emerging evidence indicates that m^7^G‐3′‐tiRNA Lys(TTT) (mtiRL), a novel m^7^G‐modified tRNA‐derived small RNA (tsRNA) catalysed by METTL1, plays an oncogenic role in BCa in vitro and in vivo. Specifically, METTL1, the tRNA m^7^G‐modifying enzyme, catalyses the formation of mtiRL, which selectively binds to the oncoprotein annexin A2 (ANXA2). This binding promotes the Tyr24 phosphorylation of ANXA2 by enhancing its interaction with Yes proto‐oncogene 1 (Yes1). Consequently, the phosphorylated ANXA2 (p‐ANXA2‐Y24) is more likely to translocate to the nucleus in BCa cells, thereby augmenting the proliferation and migration of these cells.^85^ In summary, this study reveals a novel mechanism of tsRNA epigenetic regulation in BCa malignancy, and METTL1, a tRNA m^7^G‐modifying enzyme, plays a considerable role in BCa treatment.

##### Prostate cancer

PCa is the second leading cause of cancer‐related death in men.^66^ Conventional therapies target the hormonal axis of the disease with more than 30% of patients eventually developing resistance to therapy and metastasis, highlighting the need to identify potential alternative and targetable molecular pathways for PCa treatment.^86^ One study demonstrated that METTL1 is the main epigenetic transcriptome regulatory factor in primary and advanced prostate tumours and that its overexpression is associated with poor prognosis. Specifically, METTL1‑mediated methylation protects tRNAs from cleavage into small noncoding RNAs, while METTL1 depletion causes the loss of m^7^G tRNA methylation and promotes the biogenesis of a novel class of small noncoding RNAs derived from 5′‐tRNA fragments.^61^ 5′‐tRNA‐derived small RNAs play critical roles in activating interferon (IFN) signalling pathways, immune effector processes and catabolic processes, thus suppressing PCa cell growth. In addition, in preclinical PCa models, METTL1 knockdown increases the secretion of cytokines involved in proinflammatory activity, which polarises macrophages to the M1‐like endotype and induces the down‐regulation of anti‐inflammatory cytokines which can polarise macrophages to the M2‐like endotype, indicating that METTL1 inhibition can polarise immune cells in the tumour microenvironment (TME) toward a cytotoxic tumouricidal endotype and enhance the response to immunotherapy.^61^ Furthermore, METTL1 can add m^7^G modifications to CDK14 mRNA, increasing its mRNA stability and ultimately promoting CRPC progression.^36^ In summary, these findings reveal a therapeutically actionable role of METTL1 in PCa.

##### Adrenocortical carcinoma

ACC is a highly malignant and aggressive urologic cancer, and patients commonly present with advanced or metastatic disease at the time of initial diagnosis due to atypical incipient symptoms, with a 5‐year overall survival rate of less than 15%.^87^, ^88^ METTL1 overexpression stimulates the proliferation, migration and invasion of ACC cells, whereas METTL1 silencing significantly retards tumour growth in mouse xenograft model.^41^ In addition, the levels of infiltrating CD8^+^ T cells is greater and that of macrophages is lower in clinical ACC samples with low METTL1 expression than in those with high METTL1 expression. Collectively, these results indicate that METTL1 plays a pro‐oncogenic role in ACC progression and profoundly affects the tumour immunity of ACC.

#### 4.1.4 Haematological tumours

##### Acute myeloid leukaemia

Acute myeloid leukaemia (AML) is the most common acute leukaemia in adults, and the prognosis of elderly individuals, who account for the majority of new cases, remains poor.^66^ METTL1 is frequently overexpressed in AML and is associated with poor patient survival. METTL1 increases the abundance of m^7^G‐modified tRNAs, particularly Arg‐TCT‐4‐1, as well as the translation of mRNAs, including cell cycle regulators that are enriched in the corresponding AGA codon, altering the cell cycle and inducing oncogenic transformation.^89^ As a result, this study validates the potential therapeutic role of METTL1‐mediated tRNA m^7^G modification in AML cell translation control and tumour biology.

#### 4.1.5 Head and neck tumours

##### Head and neck squamous cell carcinoma

Head and neck squamous cell carcinoma (HNSCC) is one of the most common cancers and develops from the mucosal epithelium in the oral cavity, pharynx and larynx.^66^ There is a clinical need to develop effective therapeutic regimens for HNSCC patients including molecular targeted therapy. A recent study revealed that METTL1/WDR4 accelerates HNSCC progression and metastasis and is associated with a poor prognosis. Up‐regulation of METTL1 affects the m^7^G levels of 16 tRNAs, promoting the translation of a subset of oncogenic transcripts, which includes genes related to the PI3K/AKT/mTOR signalling pathway.^90^ Chemical modulators of the PI3K/AKT/mTOR signalling pathway have also been shown to counteract the effects of METTL1 in mouse HNSCC. In addition, single‐cell RNA sequencing revealed that METTL1 knockout in mouse tumour cells alters the immune landscape and cell‐cell interactions between the tumour and stromal compartments. In conclusion, this work provides novel insights into tRNA modification‐mediated regulation of mRNA translation and highlights the critical function of METTL1 in HNSCC progression.

##### Nasopharyngeal carcinoma

More than 70% of NPC cases occur in East Asia and Southeast Asia, with terminal patients having poor prognoses, and treatment options are very limited for those patients after disease progression.^91^, ^92^ These findings emphasise the strong need for further investigations to identify potential targets of NPC therapies. Recent research has demonstrated that overexpression of METTL1 promotes NPC growth and metastasis, which is associated with poor prognosis. METTL1‐mediated m^7^G tRNA modification activates the WNT/β‐catenin signalling pathway in NPC to increase the translation of cyclins and associated oncogenes, including cyclin D1 and c‐Myc, thereby promoting the proliferation and migration of cancer cells.^47^ In addition, METTL1 promotes NPC cell EMT and chemoresistance to cisplatin and docetaxel through the WNT/β‐catenin signalling pathway. Collectively, these results indicate that METTL1 could serve as both a therapeutic target and a marker for individualised chemotherapy strategies for NPC.

##### Oral squamous cell carcinoma

OSCC is the most lethal form of HNSCC worldwide, leading to a poor prognosis because of its high recurrence and metastasis rates in advanced stages.^93^ Most patients eventually acquire resistance to anlotinib, an oral multitarget TKI that is potently antitumourigenic.^94^ METTL1 up‐regulation decreases the antiproliferative and proapoptotic effects of anlotinib while enhancing cell migration and has a markedly inverse correlation with tumour growth inhibition rates. Mechanistically, the overexpression of METTL1 in anlotinib‐resistant OSCC cells contributes to increased global mRNA translation and drives a metabolic reprogramming shift from glycolysis to OXPHOS through m^7^G tRNA modification, and the inhibition of OXPHOS biochemically negates the effect of METTL1 on anlotinib resistance.^46^ Overall, this study underscores the pivotal role of METTL1 in anlotinib resistance and lays the groundwork for novel therapeutic interventions to counteract resistance in OSCC.

##### Ameloblastoma

Ameloblastoma (AM) is the most prevalent epithelial odontogenic tumour within the oral and maxillofacial region, accounting for approximately 1% of all oral tumours, and the high recurrence rate of AM is closely related to its invasive growth behaviour.^95^ High METTL1 expression is strongly associated with postoperative recurrence in AM, and knocking down METTL1 inhibits the proliferation, migration and invasion of AM cells both in vitro and in vivo. Research has revealed that down‐regulation of METTL1 results in decreased translation levels of downstream genes from the MAPK signalling pathway, such as cyclin D1, matrix metalloproteinase‐2 (MMP2), MMP9 and vimentin, further confirming the regulatory role of METTL1 in the invasive growth of AM.^96^ These results suggest that METTL1 promotes the invasive development of AM and that the expression level of METTL1 has potential application value for predicting the prognosis of AM.

#### 4.1.6 Gynaecologic tumours

##### Breast cancer

BC is the most frequently diagnosed cancer in women worldwide, and its incidence rate has been increasing steadily.^97^ Recent research suggests that patients with luminal‐type BC may benefit from CDK4/6 inhibitor therapy, but predictive targets for guiding treatment selection are lacking.^98^ METTL1 and WDR4 overexpression in BC is correlated with poor patient prognosis, underscoring their importance in BC development. More than 50% of genes co‐expressed with METTL1 and WDR4 exhibit up‐regulated expression patterns in BC, with functional enrichment in critical cellular processes including cell cycle regulation, DNA replication and nucleocytoplasmic transport mechanisms, all of which contribute to BC progression and metastatic dissemination.^99^ Contrary to these observations, our comprehensive investigation employing m^7^G tRNA MeRIP‐seq and ribosome profiling sequencing (Ribo‐seq) revealed that METTL1‐mediated tRNA m^7^G modification exerts inhibitory effects on BC cell cycle progression and proliferative capacity. Mechanistically, METTL1 increases the m^7^G levels of 19 tRNAs to modulate the translation of GADD45A and RB1, which interact with CDKs and cyclin B1 (CCNB1), thereby leading to G2/M phase cell cycle arrest in BC cells. Furthermore, our data indicated that overexpression of METTL1 enhances abemaciclib, a CDK4/6 inhibitor, which targets the RB1 tumour suppressor protein and causes G1/S phase cell cycle arrest in tumour cells, and the combined treatment of abemaciclib with METTL1 lentivirus (LV‐METTL1) is more effective at inhibiting tumour growth than either treatment alone.^38^ These findings confirmed that METTL1 plays a critical role in BC and provide a promising strategy for improving the therapeutic benefits of CDK4/6 inhibitors in the treatment of BC patients.

##### Ovarian cancer

Ovarian cancer (OC) is a malignant gynaecological disease of the female reproductive system and is the sixth most common cause of death in women.^66^ Owing to its strong association with poor prognosis in OC, METTL1, as a differentially expressed RNA‐modification regulatory gene, was chosen to construct a signature model with LASSO regression analysis, and its risk score was negatively correlated with that of METTL1. Furthermore, the risk score was positively correlated with CD4^+^ memory resting T cells and negatively correlated with M1 macrophages and plasma cells.^100^ It is an independent prognostic model for patient stratification, prognostic evaluation and prediction of response to immunotherapy. Although this study emphasised the important roles of METTL1 in OC pathological features and prognosis, no specific experimental evidence has demonstrated the association between METTL1 and OC malignant behaviours. Taken together, these results indicate that further in‐depth research is needed to identify the functional role of METTL1 in OC progression.

##### Teratoma

Deng et al.^50^ reported that METTL1‐knockdown hiPSCs tend to differentiate into mesoderm with neovascularisation while inhibiting neuroectoderm differentiation. Increased angiogenesis is often associated with increased cancer cell proliferation and tumour growth.^101^ In nude mice, METTL1‐KD hiPSCs clearly enhance teratoma formation by promoting cell proliferation and angiogenesis by decreasing the mRNA levels of the stem cell transcription factors recombinant octamer binding transcription factor 4 (Oct4), Nanog and sex‐determining region Y (Sox2), providing novel insight into the potential role of METTL1 in teratoma treatment.^50^

#### 4.1.7 Neurological tumours

##### Glioma

Glioma, which is derived from glial cells, is the most frequently diagnosed malignant tumour of the central nervous system and accounts for more than half of all malignant brain tumours.^102^ Glioma is characterised by a high recurrence rate and mortality, and the treatment efficacy of current treatment strategies has not been satisfactory. METTL1 expression continuously increases with increasing glioma grade and was significantly greater in glioma tissues than in adjacent noncancerous tissues. In addition, the results of functional enrichment and pathway analyses indicate that the molecular mechanism by which high METTL1 promotes glioma proliferation is potentially related to the MAPK signalling pathway, and further study of the relationship between METTL1 and this pathway is necessary.^103^ Furthermore, high METTL1 expression is associated with poor prognosis and survival of patients with glioma. METTL1 may be used as a valuable independent risk factor, and further investigations are warranted to explore its clinical application.

##### Neuroblastoma

Neuroblastoma (NBL) is the most universal malignant tumour in infancy and the most common extracranial solid tumour in childhood, with extremely low survival for advanced NBL.^104^ A recent study revealed that METTL1 is noticeably up‐regulated in advanced NBL and plays a crucial role in promoting NBL progression, whereas down‐regulation of METTL1 inhibits cell proliferation and decreases the migration ability of NBL cells. Furthermore, m^7^G profiling and translation analysis demonstrated that METTL1‐mediated tRNA m^7^G modification crucially selectively regulates NBL cell mRNA translation in a m^7^G‐related codon‐dependent manner. Knockdown of METTL1 reduces the levels of both m^7^G modification and m^7^G tRNA expressions, with impaired translation of 339 overlapping genes enriched in oncogenic pathways in NBL cells, including metadherin (MTDH, also known as AEG‐1 and Lyric) and programmed cell death 10 (PDCD10).^105^ In summary, this study reveals the tumour‐promoting role of METTL1‐mediated tRNA m^7^G modification in controlling NBL progression, providing a potential strategy for the clinical management of NBL.

#### 4.1.8 Musculoskeletal tumours

##### Osteosarcoma

Osteosarcoma is one of the most common primary bone tumours and is associated with a high mortality rate in children and adolescents, and surgery combined with adjuvant chemotherapy is the most effective treatment for osteosarcoma patients.^106^, ^107^ Owing to its high metastasis and drug resistance rates, the clinical outcomes for osteosarcoma patients have not improved.^108^ METTL1 is frequently amplified in osteosarcoma and is associated with poor patient prognosis. METTL1 knockdown decreases the tRNA m^7^G modification level and impairs osteosarcoma progression, whereas METTL1 overexpression promotes osteosarcoma proliferation, migration and invasion. In detail, m^7^G dysregulation of tRNAs mediated by METTL1 increases the translation of genes associated with extracellular matrix remodelling, an important factor contributing to chemoresistance in cancers, including LOXL2, which facilitates osteosarcoma progression and chemoresistance to doxorubicin.^42^ However, another study revealed that METTL1 expression is markedly lower in osteosarcoma tissues than in normal bone tissues and that METTL1 expression decreases with increasing clinical stage. Furthermore, METTL1 promotes primary pri‐miR‐26a maturation by increasing RNA stability in a manner dependent on m^7^G methylation. The increase in mature miR‐26a‐5p can further target FTH1 mRNA and eliminate FTH1 translation efficiency, increasing ferroptosis and promoting the sensitivity of osteosarcoma cells to chemotherapeutic drugs.^54^ Thus, more experimental evidence is needed to clarify the specific function of METTL1 in osteosarcoma.

### 4.2 The role of METTL1 in other diseases

**FIGURE 6:**
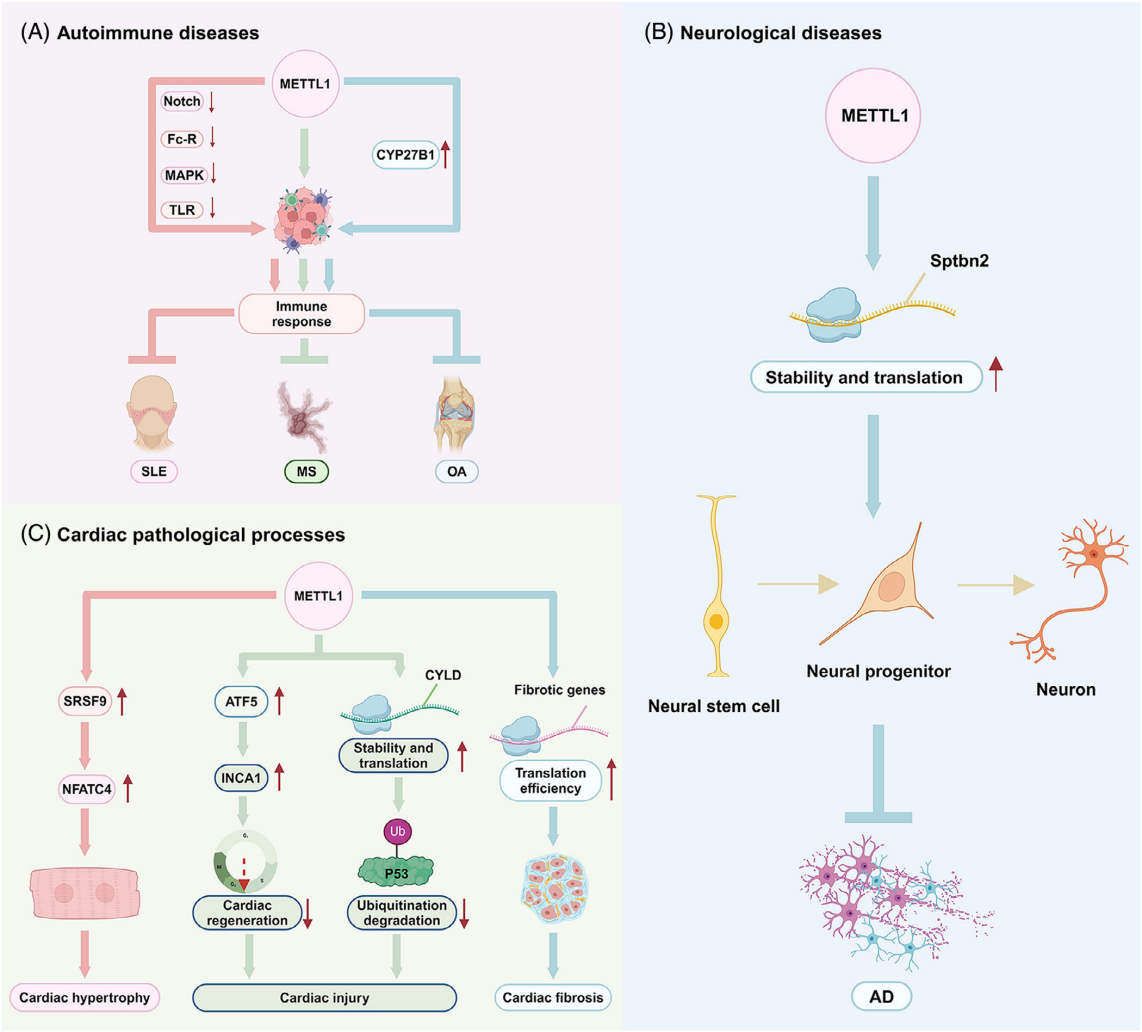
The role of METTL1 in autoimmune diseases, neurological diseases and cardiac pathological processes. METTL1 regulates the progression of diseases other than tumours by modulating the expression of related genes, encompassing autoimmune diseases such as SLE, MS and OA (A), neurological diseases like AD (B) and cardiac pathological processes including cardiac hypertrophy, cardiac injury and cardiac fibrosis (C).

Several studies have demonstrated that METTL1 is closely associated with the initiation, progression and prognosis of diseases other than tumours. METTL1 is commonly aberrantly expressed in diseases and regulates disease progression (Table 3 and Figure 6). Here, we have summarised the role of METTL1 in the progression of other diseases.

**TABLE 3:** Regulation of METTL1 in other disease progression.

Disease type	Expression level	Roles	Functions	Mechanism	References
SLE	Down	Suppressor	Regulated immune response	Regulated immune cells; inhibited the Notch, Fc receptor‐mediated phagocytosis, MAPK and toll‐like receptor signalling pathway	^65^
MS	No study	Suppressor	Regulated immune response	Activated CYP27B1 and regulated vitamin D metabolism	^118^, ^119^
OA	Down	Suppressor	Regulated immune response	Associated with immune cell infiltration	^122^
AD	Down	Suppressor	Promoted neurogenesis	Enhanced the stability and translation of Sptbn2 mRNA	^125^
Cardiac hypertrophy	Up	Promoter	Promoted cardiac remodelling	Increased SRSF9 expression and promoted alternative splicing and stability of NFATc4	^62^
Cardiac injury	Up	Promoter	Inhibited cardiomyocyte proliferation and cardiac regeneration	Increased ATF5 expression and promoted the transcription of INCA1 Increased CYLD expression and enhanced the stability of P53	^37^, ^129^
Cardiac fibrosis	Up	Promoter	Promoted cardiac fibroblast proliferation and myofibroblast transformation	Increased fibrotic genes and enhanced their translation efficiency	^132^

#### 4.2.1 Autoimmune diseases

##### Systemic lupus erythematosus

SLE is caused by pathogenic autoantibodies and has diverse clinical manifestations caused by the phenotypic and functional abnormalities of immune cells.^109^ Conventional treatments are often accompanied by unwanted side effects such as kidney damage and infection, which seriously impair the quality of life of SLE patients, and targeted and individualised therapy should be further developed.^110^ One study suggested that METTL1 is down‐regulated in patients with SLE and that METTL1 is positively correlated with the number of peripheral blood B cells but negatively correlated with the number of suppressor T cells, indicating that METTL1 deficiency has the potential to participate in the progression of SLE.^65^ Furthermore, METTL1 expression is negatively correlated with aberrant immune pathways, including the Notch signalling pathway, Fc receptor‐mediated phagocytosis, the MAPK pathway and the Toll‐like receptor signalling pathway, which are reportedly to be involved in SLE by regulating immune cells or inflammatory cytokines.^65^, ^111^, ^112^, ^113^, ^114^ In addition, METTL1 is positively correlated with activated CD8 T^+^ cells and activated CD4^+^ T cells, which are abnormally activated because of aberrant T‐cell signalling pathways, resulting in inflammatory responses, which facilitate B cells and autoimmunity.^65^, ^115^ These results indicate that METTL1 might influence the immune response by affecting the dysregulation of immune cells in SLE, which might be explored for a more effective immunotherapy for SLE.

##### Multiple sclerosis

Multiple sclerosis (MS) is a chronic autoimmune disease with a complex pathogenesis in which neurodegeneration and demyelination are the main contributors to disability.^116^ Tag‐single nucleotide polymorphism analysis in MS patients revealed that a functional variant (rs10877013) strongly affects the activity of one enhancer in the 12q13.3–12q14.1 region in an allele‐dependent and orientation‐dependent manner, which could be the cause of the alteration in KIF5A–CYP27B1–METTL1–FAM119B locus gene expression due to the promoter multigene interactions observed in the region.^117^ In addition, MS patients have lower levels of vitamin D in their serum, which has a suppressive role in the adaptive immune system. CYP27B1, as an enzyme induced by PTH and implicated in vitamin D activation, could be the causal gene.^118^ It has been proposed that deletion of METTL1 results in loss of PTH activation of CYP27B1, implying a link between METTL1 and the pathogenesis of MS.^119^

##### Osteoarthritis (OA)

Osteoarthritis (OA) is the most common form of arthritis and has a poor prognosis, affecting more than 7% of the global population.^120^ Unfortunately, there is a lack of satisfactory treatments for effectively treating OA patients in the clinical.^121^ Analysis revealed that METTL1 expression is lower in OA samples than in healthy samples and is strongly correlated with immune cells and immunological function. METTL1 was identified as a novel m^7^G hub biomarker through an intersection analysis during the progression of OA and was used to construct a model to predict the occurrence of OA.^122^ This study highlights the importance of METTL1 in OA, which contributes to enhancing our understanding of immune infiltration characterisation and guides the development of more effective immunotherapy strategies.

#### 4.2.2 Neurological diseases

##### Alzheimer's disease

Alzheimer's disease (AD) is the most common irreversible type of dementia characterised by cognitive dysfunction and marked neuronal loss, which is attributed to adult hippocampal neurogenesis impairment.^123^, ^124^ The expression of METTL1 and the m^7^G modification level in neurons are considerably increased compared with those in neural stem cells, and METTL1 overexpression enhances neuronal differentiation and proliferation. Mechanistically, METTL1‐mediated internal m^7^G modification facilitates SPTBN2 expression by increasing SPTBN2 mRNA stability and translation, hence promoting neurogenesis. In addition, METTL1 knockdown reduces hippocampal neurogenesis and spatial memory, whereas METTL1 overexpression rescues defective neurogenesis and cognitive impairment in adult mice.^125^ These results suggest that targeting METTL1‐mediated regulation of neurogenesis could be a potential therapeutic option for the treatment of AD.

#### 4.2.3 Cardiac pathological processes

##### Cardiac hypertrophy

Cardiac hypertrophy is a compensatory response of the myocardium to mechanistic stress provoked by various cardiac disorders, and pathological cardiac hypertrophy stemming from persistent stress is an independent risk factor for the development of heart failure (HF).^126^ Deficiency of METTL1 attenuates cardiac hypertrophy and dysfunction upon pressure overload from transverse aortic constriction or angiotensin II stimulation, whereas cardiac‐specific overexpression of METTL1 drives cardiac remodelling. Mechanistically, METTL1 increases SRSF9 expression by inducing m^7^G modification of SRSF9 mRNA, which enhances alternative splicing and the stability of NFATc4, ultimately leading to a prohypertrophic phenotype and facilitating hypertrophic cardiac growth.^62^ This study reveals that METTL1 plays an important role in driving cardiac hypertrophy by regulating the SRSF9/NFATc4 axis, suggesting that targeting METTL1 could be an efficient way to treat cardiac hypertrophy and HF.

##### Cardiac injury

Myocardial infarction leads to functional cardiac cell injury, which is the basic pathological process that induces HF.^127^ METTL1 has been shown to increase m^7^G methylation of ATF5 mRNA and increase ATF5 expression, thereby promoting the transcription of INCA1, an inhibitor of CDK2 that interacts with cyclin A1, resulting in S to G2/M cell cycle arrest and suppressing cardiomyocyte proliferation and cardiac regeneration.^37^, ^128^ Moreover, METTL1 improved the deubiquitinase cylindromatosis (CYLD) mRNA stability by inducing m^7^G modification of CYLD mRNA, thereby leading to increased CYLD protein expression. This process results in an increase in the CYLD‐mediated deubiquitination of P53 and ultimately leads promotes cardiomyocyte apoptosis in cardiac ischemia/reperfusion injury.^129^ Hence, these findings reveal that METTL1‐mediated m7G methylation is involved in the regulation of cardiomyocyte proliferation, opening new avenues for alleviating cardiac injury and promoting cardiac repair and regeneration.

##### Cardiac fibrosis

Cardiac fibrosis is a common pathology of heart disease that damages the heart physically and electrically.^130^, ^131^ Therefore, identifying potential targets for the treatment of cardiac fibrosis is crucial. A recent study demonstrated that METTL1‐mediated RNA m^7^G methylation is elevated in cardiac fibrosis tissues and that TGF‐β1‐induced myofibroblast transformation of cardiac fibroblasts is an important step in the development of cardiac fibrosis. Furthermore, fibroblast‐specific knockout of METTL1 decreases the number of m^7^G‐methylated fibrotic genes and impairs their translation efficiency, thereby attenuating MI‐induced HF and cardiac fibrosis.^132^ Overall, these findings suggested a novel profibrosis role of METTL1‐mediated m^7^G methylation and that targeting METTL1 could be a potential and attractive therapeutic strategy for cardiac fibrosis.

#### 4.2.4 Other diseases

Fu et al. elucidated the essential role of METTL1‐mediated tRNA m^7^G modification in mediating protein synthesis and protein homeostasis, thereby regulating senescence. METTL1 is down‐regulated during cell senescence and aging at both the transcriptional and protein levels, resulting in a subset of tRNAs being targeted for rapid tRNA degradation due to m^7^G46 hypomethylation. The decreases lead to ribosomes stalling at certain codons, suppressing globe translation initiation and elongation that are essential in pathways such as WNT signalling and ribosome biogenesis. Furthermore, chronic ribosome stalling stimulates the ribotoxic stress responses and integrated stress responses, which activate senescence‐associated secretory phenotype.^133^ A recent study unveiled a critical role of METTL1 in bone development. Loss of METTL1 decreases abundance of m^7^G‐modified tRNAs and selectively inhibits translation of mRNAs relating to cytoskeleton and Rho GTPase signalling, which up‐regulates the level of branched‐chain amino acid transaminase 1 that rewires cell metabolism and depletes intracellular α‐ketoglutarate (αKG). Restoring αKG can promote mineralisation and calcium deposition of skeletal stem cells and alleviate the skeletal defect, indicating that METTL1 might be a therapeutic option for primordial dwarfism.^134^ Moreover, Mettl1, the Drosophila ortholog of the m^7^G‐modifier METTL1, is required for Drosophila fertility. Depletion of Mettl1 results in a loss of m^7^G modification and decreases tRNA abundance. Consistent with the decrease in tRNA abundance, Ribosome profiling shows increased occurrence of ribosome pausing in Mettl1‐KO testes at the codons decoded by the reduced tRNAs, leading to significant reduction of the translation efficiency of genes involved in elongated spermatid formation and sperm stability. The study showed the m^7^G‐modified tRNA species to be largely similar between Drosophila and mammals, which implicates that METTL1 might play a similar role in human gonadal tissues.^135^

## 5 TARGETING METTL1 FOR POTENTIAL CLINICAL APPLICATION

On the basis of its diverse functions and molecular mechanisms in disease, targeting METTL1 may provide a novel perspective for individualised disease therapy. Moreover, the development of METTL1 agonists and inhibitors is theoretically feasible because of increasingly clear structural and functional characteristics. The functional domain of METTL1 can be considered a target of inhibitors. The N‐terminus of METTL1 couples cofactor SAM binding, with key conformational changes in the tRNA, the catalytic loop and the C‐terminal tail of WDR4 acting as a switch to activate m^7^G methylation.^31^ The competitive binding of small‐molecule complexes or modification of the N‐terminal region of METTL1 (serine27 phosphorylation) can effectively reduce methyltransferase activity by locally disrupting the catalytic centre.^136^ Similarly, we can assume that a small molecule may improve the activity of the METTL1/WDR4 complex and that the compound interacts with SAM in close proximity to the active centre of METTL1, potentially increasing the binding affinity of SAM and lowering the energy barrier of the substrate RNA methylation reactions. Similar drug development strategies have shown preliminary success with other RNA modification proteins, such as METTL3, and are ready to enter clinical trials.^137^, ^138^ Although METTL1 inhibitors have not yet been developed, we can consider screening potential candidates from the United States Food and Drug Administration‐approved small molecule inhibitor library or traditional Chinese medicine database. A growing body of evidence highlighting the critical role of METTL1 in diseases may lead to new possibilities for treating diseases with inhibitors or activators of METTL1. For example, central to the efficacy of immune checkpoint blockade therapy is the requirement for cytotoxic immune cells to infiltrate tumours, whereas specifically inhibiting METTL1 exactly leads to enhanced infiltration of cytotoxic macrophages and cytotoxic T cells into tumours, indicating that METTL1 inhibitors are emerging as immune modulators of the TME and combining METTL1 inhibitors with immunotherapy may have a synergistic effect on increasing the efficacy of PCa treatment.^61^ Our study revealed that combining a lentivirus targeting METTL1 with abemaciclib is more effective at inhibiting tumour growth than either treatment alone is, suggesting that developing agonists for METTL1 could be a promising therapeutic strategy to increase the efficacy of CDK4/6 inhibitors in treating BC.^38^

## 6 CONCLUSION AND PERSPECTIVES

As one of the most prevalent post‐transcriptional modifications of RNA, m^7^G methylation has gradually become a hot topic in cancer research, but the role of the methyltransferase METTL1 in this process is still at an early stage. We have demonstrated that METTL1 is a double‐edged sword for diseases and summarised its biological functions by affecting the m^7^G modifications of various RNAs. METTL1 has been found to accelerate cancer cell proliferation, invasion and metastasis in a number of cancers while restraining the development of certain types of tumours, such as CC, LC, BC, OC, teratoma and osteosarcoma.^24^, ^38^, ^50^, ^54^, ^64^, ^100^ In LC, BC and CC, METTL1 has been reported to have either oncogenic or tumour suppressive functions in different studies, which could be explained by tumour heterogeneity or the different model systems used in these studies, and further comprehensive and detailed studies are warranted to gain a better understanding. In addition, METTL1 can regulate glycolipid and mitochondrial metabolism in ACC and OSCC.^41^, ^46^ An essential role of METTL1 in cancer cell escape from chemotherapy and radiotherapy has also been reported in various tumours. METTL1 enhances OSCC resistance to anlotinib, whereas METTL1 up‐regulation increases CC sensitivity to cisplatin.^46^, ^64^ Moreover, METTL1 triggers resistance to the drug lenvatinib in HCC and promotes resistance to IR in HCC cells.^55^ Interestingly, one study revealed that METTL1 enhances osteosarcoma chemoresistance to doxorubicin,^42^ whereas another study reported that METTL1 enhances ferroptosis in osteosarcoma and increases the sensitivity of osteosarcoma cells to chemotherapy drugs.^54^ Furthermore, METTL1 is a promising novel target for reshaping the TIME and offers a potential strategy for PCa and ACC immunotherapy.^41^, ^61^ Co‐blockade of METTL1 and its downstream chemokine pathway is a mechanism‐based combination strategy that improves anti‐PD‐1 efficacy in ICC.^59^ In addition to its effect on tumours, METTL1 also has a considerable suppressive effect on autoimmune diseases by regulating immune cell infiltration and the immune response. Moreover, METTL1 regulation of neurogenesis may be explored for the developing of targeted molecular therapies for AD. METTL1‐catalysed RNA m^7^G modification has been implicated in cardiac hypertrophy, cardiac injury and cardiac fibrosis, highlighting its potential as a therapeutic target in cardiac pathological processes. Taken together, these findings suggest that METTL1 is closely related to and plays vital roles in various diseases and may serve as a novel target for disease treatment; however, certain precise mechanisms remain unknown.

Given the significant role of METTL1‐mediated m^7^G modification in many diseases, rational development and utilisation of RNA modification detection and sequencing techniques can rapidly identify m^7^G modification present in various model organisms or cell types and map it to different types of RNA. m^7^G–MeRIP‐seq is a high‐throughput sequencing method that utilises the principle of antibody‐specific binding to methylated bases. It enriches m^7^G‐modified RNA fragments by immunoprecipitation with specific antibody against m^7^G methylation.^139^ Specifically, the RNAs were interrupted into short fragments and incubated the methylation modified fragments with anti‐m^7^G antibody. Then, the m^7^G RNA methylome was reconstructed for sequencing and data analysis.^140^ This technique is convenient, fast and allows for a qualitative analysis of highly methylated RNA regions. However, it is largely limited by specific antibodies and cannot achieve single‐base resolution in identifying m^7^G methylation. m^7^G AlkAniline‐seq can precisely reverse this disadvantage by employing chemical methods and deep sequencing technologies to detect m^7^G methylation in RNAs at single‐base resolution with high sensitivity and specificity.^141^ Moreover, TRAC‐seq, a chemical‐based approach of combining the AlkB‐mediated tRNA‐sequencing with sodium borohydride/aniline‐induced cleavage at m^7^G sites to achieve single‐base resolution profiling of m^7^G modifications, which is currently the most widely used technology for m^7^G research.^142^, ^143^ Based on the principle of the TRAC‐seq method, this method could be extended to other chemical cleavage technologies that generate 5′‐phosphate ends for single‐base resolution analysis in various species of RNAs.^144^ We expect that these existing detection schemes of RNA methylation can assist researchers in overcoming difficulties and developing new therapies for diseases targeted at METTL1‐mediated m^7^G modification.

Unfortunately, METTL1 has not been targeted successfully with effective therapeutic drugs, limiting the immediate translational value of targeting METTL1. Recent studies have developed small‐molecule and proteolysis‐targeting chimera (PROTAC) inhibitors for multiple other RNA modification enzymes, such as METTL3.^138^, ^145^ Nai et al.^146^ discovered a series of small‐molecule inhibitors of METTL1 by an enzymatic assay based on luminescence and high‐throughput docking. Such enzymatic scintillation assays based on detecting enzyme activity are valuable for the determination of inhibitory constants (IC50 and KI) of advanced drugs and lead‐like candidate drugs but have notable shortcomings compared with biophysical ligand binding assays, such as an increase in susceptibility to assay interference and poor reproducibility at high ligand concentrations.^147^ Besides these limitations, the time consumption and the instrument/reagent costs of current enzyme activity‐based and biophysical methyltransferase assays are already high, limiting the use of medium to high‐throughput applications and hindering the steadily evolving field of medicinal chemistry related to METTL1‐mediated m^7^G modification.^148^ With an increasing understanding of the functions, regulation and mechanisms of METTL1 in disease, we expect that METTL1‐targeting agents will be developed in the near future to potentiate the therapeutic effect of treating human diseases. However, it is undeniable that several issues remain to be addressed. First, the development of METTL1 inhibitors, which are important protein molecules in cells, must consider their impact on normal cellular functions. As a result, the safety and tolerability of METTL1 inhibitors must be thoroughly examined during the drug development process. Second, single‐target therapy frequently fails to provide optimal therapeutic effects owing to the complexity and heterogeneity of cancer. Thus, the comprehensive effects of multiple factors need to be considered when assessing the efficacy of METTL1 inhibitors. Third, owing to the translation process from basic research to clinical application involving numerous phases and a lengthy verification period, the clinical translation of METTL1 requires patience and ongoing efforts. As discussed above, the therapeutic translation of METTL1 is a field full of challenges and opportunities. With ongoing studies and technology improvements, METTL1 may be an important target for future human disease treatment.

In conclusion, METTL1 affects a broad range of biological processes and plays an indispensable role in cancers and other diseases, but targeting METTL1 for clinical application is still in its infancy. Many studies have revealed that METTL1 may be used as a potential biomarker to predict disease development, opening new avenues for disease‐targeted therapy. Thus, further studies are warranted to fully elucidate the mechanisms by which METTL1 affects various diseases, and ongoing efforts should be made to determine the potential of METTL1 for therapeutics targeting human disease occurrence and development.

## AUTHOR CONTRIBUTIONS

M. Z., J. H. and F. H. provided direction and guidance throughout the preparation of the manuscript. H. F. performed the literature search and wrote the original manuscript. D. D., X. W. and X. X. provided constructive suggestions and made significant revisions to the manuscript. L. L., Y. Z. and Y. W. prepared the figures and tables. Y. L. and H. W. helped revise the manuscript. All authors read and approved the final manuscript.

## CONFLICT OF INTEREST STATEMENT

The authors declare that they have no known conflicts of interest or personal relationships that could have appeared to influence the work reported in this paper.

## ETHICS STATEMENT

Not applicable.
